# Single nucleotide polymorphisms generated by genotyping by sequencing to characterize genome-wide diversity, linkage disequilibrium, and selective sweeps in cultivated watermelon

**DOI:** 10.1186/1471-2164-15-767

**Published:** 2014-09-08

**Authors:** Padma Nimmakayala, Amnon Levi, Lavanya Abburi, Venkata Lakshmi Abburi, Yan R Tomason, Thangasamy Saminathan, Venkata Gopinath Vajja, Sridhar Malkaram, Rishi Reddy, Todd C Wehner, Sharon E Mitchell, Umesh K Reddy

**Affiliations:** Gus R. Douglass Institute, Department of Biology, West Virginia State University, Dunbar, WV 25112-1000 USA; U.S. Vegetable Laboratory, USDA, ARS, 2875 Savannah Highway, Charleston, SC 29414 USA; Department of Horticultural Science, North Carolina State University, Raleigh, NC 27695-7609 USA; Genomic Diversity Facility, Institute of Biotechnology, Cornell University, Ithaca, NY 14853 USA

**Keywords:** Linkage disequilibrium, GWAS, Selective sweep, Population structure, Genotyping by sequencing, Watermelon, *Citrullus lanatus* var. *lanatus*

## Abstract

**Background:**

A large single nucleotide polymorphism (SNP) dataset was used to analyze genome-wide diversity in a diverse collection of watermelon cultivars representing globally cultivated, watermelon genetic diversity. The marker density required for conducting successful association mapping depends on the extent of linkage disequilibrium (LD) within a population. Use of genotyping by sequencing reveals large numbers of SNPs that in turn generate opportunities in genome-wide association mapping and marker-assisted selection, even in crops such as watermelon for which few genomic resources are available. In this paper, we used genome-wide genetic diversity to study LD, selective sweeps, and pairwise *F*_*ST*_ distributions among worldwide cultivated watermelons to track signals of domestication.

**Results:**

We examined 183 *Citrullus lanatus* var. *lanatus* accessions representing domesticated watermelon and generated a set of 11,485 SNP markers using genotyping by sequencing. With a diverse panel of worldwide cultivated watermelons, we identified a set of 5,254 SNPs with a minor allele frequency of ≥ 0.05, distributed across the genome. All ancestries were traced to Africa and an admixture of various ancestries constituted secondary gene pools across various continents. A sliding window analysis using pairwise *F*_*ST*_ values was used to resolve selective sweeps. We identified strong selection on chromosomes 3 and 9 that might have contributed to the domestication process. Pairwise analysis of adjacent SNPs within a chromosome as well as within a haplotype allowed us to estimate genome-wide LD decay. LD was also detected within individual genes on various chromosomes. Principal component and ancestry analyses were used to account for population structure in a genome-wide association study. We further mapped important genes for soluble solid content using a mixed linear model.

**Conclusions:**

Information concerning the SNP resources, population structure, and LD developed in this study will help in identifying agronomically important candidate genes from the genomic regions underlying selection and for mapping quantitative trait loci using a genome-wide association study in sweet watermelon.

**Electronic supplementary material:**

The online version of this article (doi:10.1186/1471-2164-15-767) contains supplementary material, which is available to authorized users.

## Background

Watermelon, ranking among the top five most-frequently purchased fruits, is cultivated globally, with a per capita annual consumption of ~7 kg (National Watermelon Promotion Board, 2010). Narrow genetic diversity is associated with susceptibility to a large number of diseases and pests among the world’s cultivated watermelons. Modern breeding practices have stressed the introgression of new genetic variation, especially for disease resistance, from underutilized germplasm accessions.

One explanation for the narrow genetic diversity in American and European germplasm could be the founder effect, whereby a small number of accessions are brought to a continent or region as people travel [1, 2]. Watermelons may have either entered Europe around 512 AD, when the Moors invaded the Iberian peninsula or during the Crusades [2]. In India and China, watermelon was introduced around 800 and 1100 AD, respectively [3]. The introduction of watermelon cultivars into the Americas occurred after the second voyage of Columbus and during the slave trade and colonization [2–4].

Nimmakayala et al. [5] performed the most recent diversity analysis of watermelon with 134 single nucleotide polymorphisms (SNPs) and 130 cultivars belonging to Africa, Asia, Europe, and the Americas. The authors found seven different clusters, with no clear distinction of accessions based on the site of collection or geographic identity. These findings agree with previous studies [4, 6–9] concluding that molecular diversity in cultivated watermelon has a range of 2–4%. The set of polymorphic SNPs previously used were too limited to address important population genetic questions necessary for association genetics.

In addition to molecular diversity, the extent of linkage disequilibrium (LD) in sweet watermelon collections must be estimated. The number of markers needed to perform genome-wide association studies (GWASs) depends largely on the extent of LD in the breeding population [10]. In other words, if the LD is large in a breeding population, a moderate number of SNPs would suffice for a GWAS. Employing genotyping by sequencing (GBS) to develop large numbers of SNPs and mapping them to a reference genome sequence created a unique opportunity to perform a GWAS in crops such as watermelon, for which few genomic resources are available [5, 11–14]. Dense genome-wide SNP datasets generated by GBS can be used to estimate chromosome-wide molecular diversity and population structure very precisely. Detecting and accounting for population stratification is essential in GWASs to reduce spurious associations [13, 15, 16].

The reduction in nucleotide diversity underlying genetic bottlenecks during domestication causes selective sweeps in genomic areas containing genes of agricultural importance [17]. Selection by early farmers and systematic breeding efforts to improve varieties can significantly affect genetic diversity, as has occurred in several crops and domestic animals [18–21]. Strong selection can fix advantageous large-effect mutations underlying domestication, ecotype characteristics, adaptation, and fruit quality for example [22]. Such selection is reflected in chromosomal regions as sweeps, whereby diversity flanking the selected allele is eroded [23]. Characterizing the genome-wide distribution of genetic diversity has identified selective sweeps in the genomes of many crop and animal species [20, 24, 25]. Detecting selective sweeps can elucidate the identities of genes and mutations with large phenotypic effects, even if they are no longer segregating within any one population. Such areas cannot be detected by forward genetics [26].

The emergence of high-throughput SNP datasets has allowed for GWASs of crop plants [27–29]. Most crops other than maize and rice feature extensive LD because of bottlenecks. In this case, a medium-resolution GWAS can still be applied to capture significant genetic effects present in the cultivated gene pool using a few thousand SNPs with a minor allele frequency (MAF) of 0.5% or greater [27]. In this study, we analyzed genome-wide diversity using a large SNP dataset from a collection of watermelon cultivars drawn from representative accessions grown across the world. We characterized genome-wide LD and explored its genetic effects on soluble solid content (SSC). We also identified and characterized regions of the watermelon genome that have been subjected to selective sweeps. We selected 183 watermelon accessions, including the previously tested 130 accessions, with 134 SNP markers [5]. This collection represents the entire world cultivars, with wide ecotype variation.

## Results

### SNP identification and characterization

Of 23,693 SNPs genotyped in this study, 11,485 were filtered with a MAF of ≥ 0.01, a call rate of 90%, and biallelic. Chromosomes 1–11 contained 1,472, 1,062, 1,062, 529, 1,357, 1,003, 887, 700, 1,171, 1,372, and 870 SNPs, respectively. Further LD pruning to remove the duplicate or non-informative markers combined with minimal MAF = 0.05 and Hardy-Weinberg equilibrium (HWE) (P > 0.01) resulted in 5,254 associations mapping suitable SNPs. We characterized 1,326 SNPs located in various exons across the whole genome, 3,928 were from the non-coding part of the genome. Non-coding SNP distribution on chromosomes 1–11 represented 395, 357, 308, 202, 430, 312, 328, 268, 395, 310, and 323 SNPs, respectively. Exon-specific SNPs on chromosomes 1–11 represented 269, 88, 73, 47, 139, 75, 67, 45, 154, 301, and 68 SNPs, respectively.

### Molecular diversity and population structure

**Figure 1:**
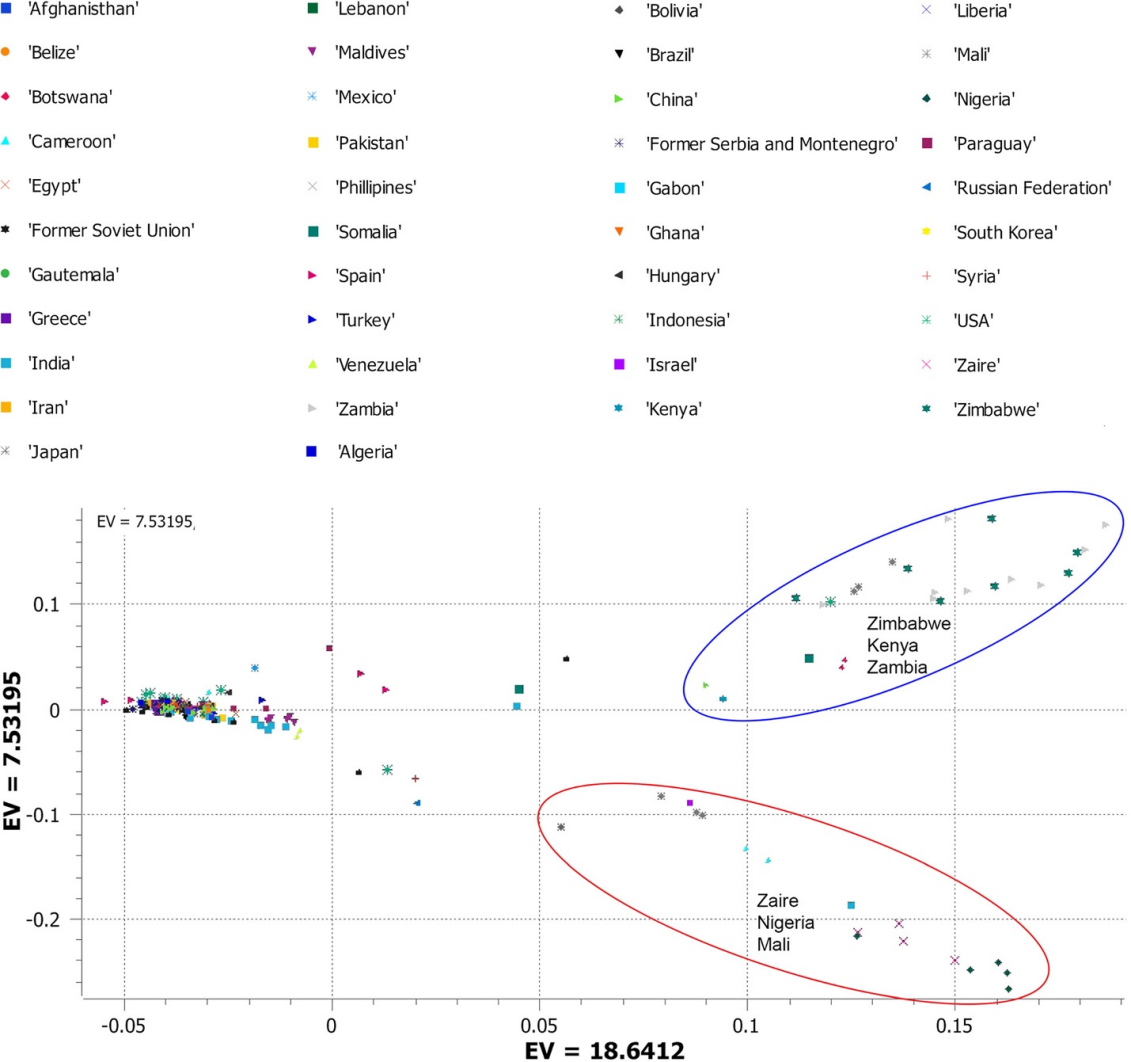
**Principal component analysis (PCA) showing the first two components of global watermelon accession collections with 5,254 single nucleotide polymorphisms (SNPs) generated by genotyping by sequencing (GBS).**

**Figure 2:**
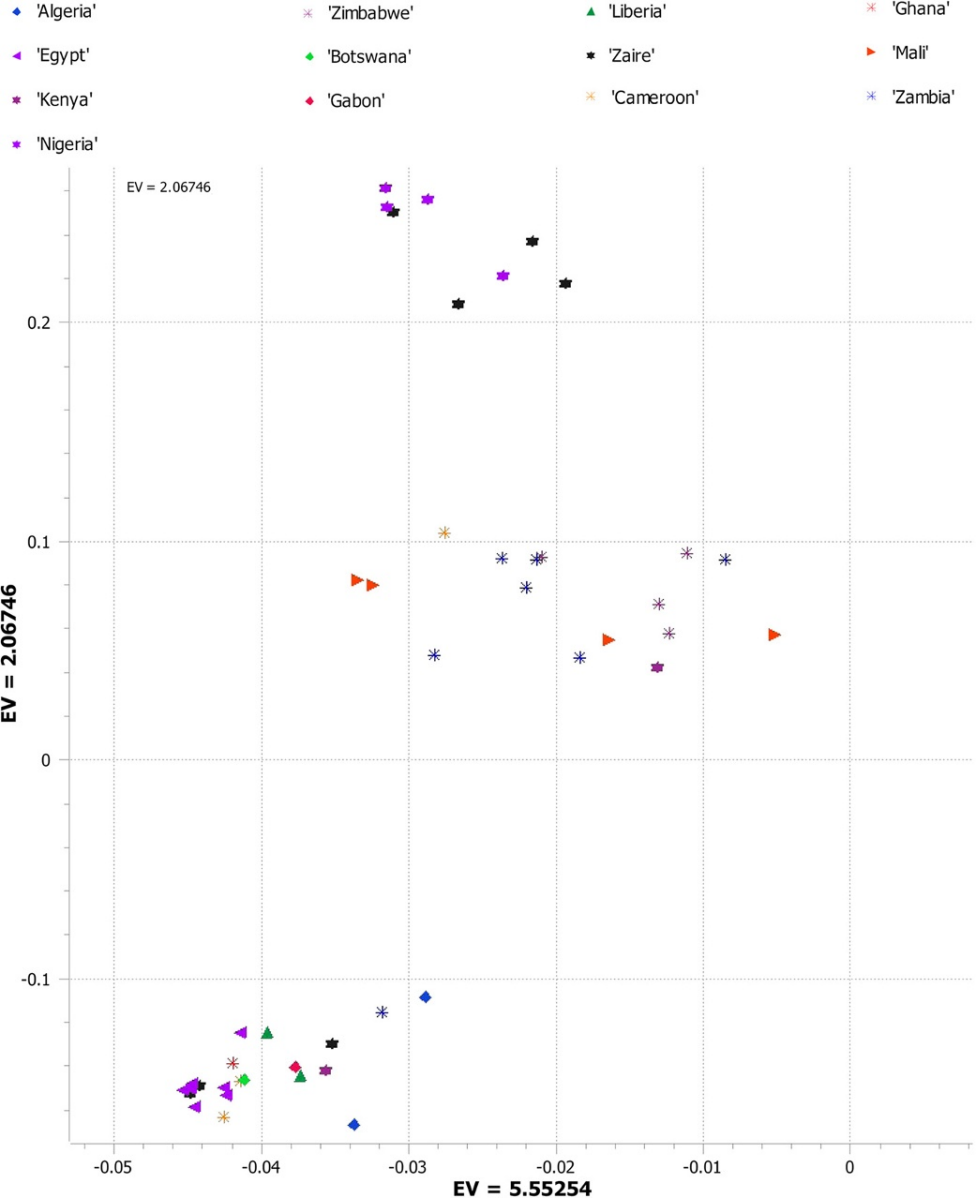
**PCA showing the first two components of Africa watermelon accession collections using 5,254 SNPs generated by GBS.**

**Figure 3:**
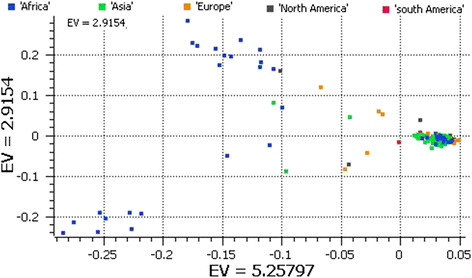
**PCA of global watermelon accession collections with 3,928 noncoding SNPs.**

**Figure 4:**
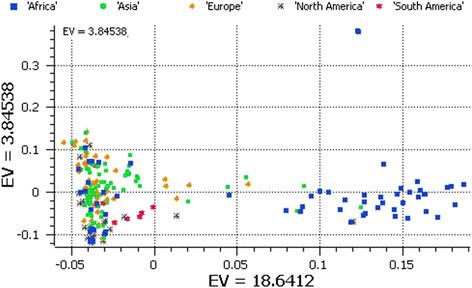
**PCA of global watermelon accession collections with 1,326 SNPs located in the exons.**

We used principal component analysis (PCA) of the 5,254 SNPs to classify sweet watermelons belonging to various countries. Accessions from Zimbabwe, Zambia, and Kenya were grouped in quadrant I and accessions from Zaire, Mali, and Nigeria in quadrant IV. The rest of the African accessions and those from various Asian, European, and American countries clustered in quadrant III (Figure 1). A second PCA performed to understand the relationships of accessions in Africa alone produced three clusters (Figure 2): the accessions from Zaire and Nigeria (top cluster in the PCA); Zimbabwe, Kenya, and Zambia (cluster located in the center of PCA); and a mixture of accessions from various African countries (the cluster in the bottom of the PCA), which was shared with the global accessions. In addition, with 3,928 SNPs located in non-coding regions, PCA grouped African sweet watermelons into two clusters as in PCA I (Figure 3). To understand the effect of coding SNPs on clustering, PCA IV grouped all subclusters of the African types, so that the exon-specific SNPs were less discriminative than the non-coding SNPs (Figure 4).

**Figure 5:**
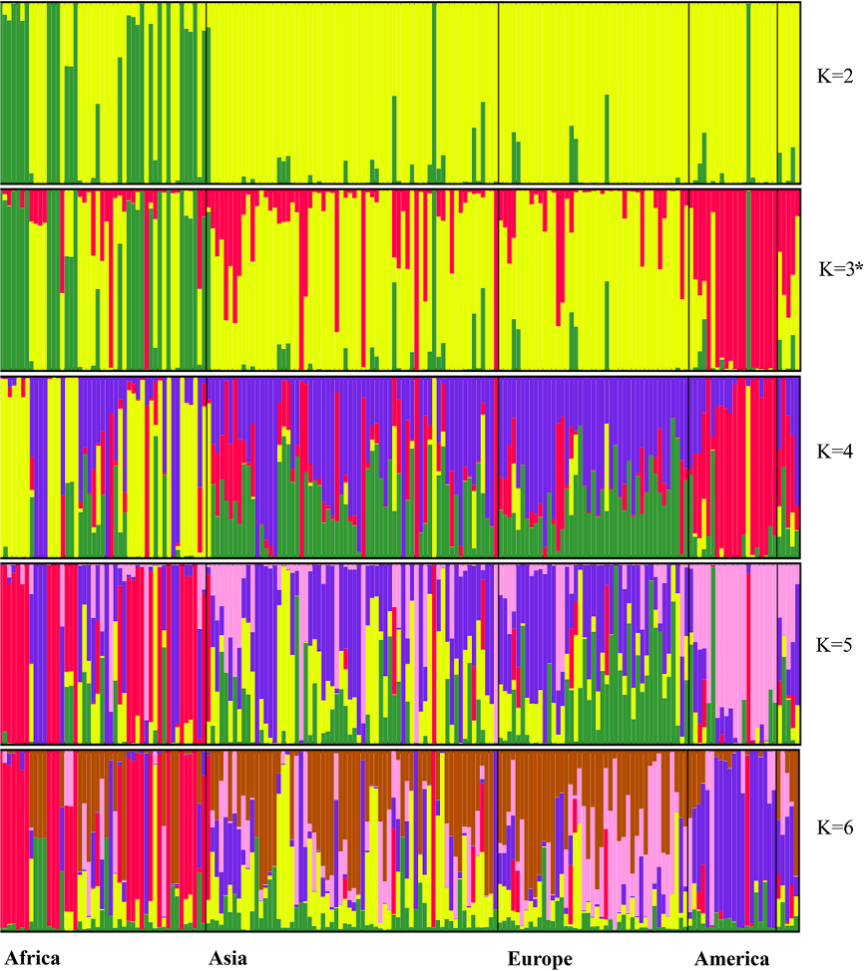
**Ancestry analysis of global watermelon accession collections by population structure, a model-based approach.** K3 had the highest peak (based on Delta K distribution) indicating that three clusters sufficiently define watermelon population structure.

We used a model-based approach to population structure analysis to analyze the entire panel of 183 sweet watermelon accessions (Figure 5). Use of Structure Harvester provided mean LnP(K) and Delta K values (Additional file 1: Figure S1). K-3 was the most appropriate cluster for this population, with the highest Delta K value of 550 as compared with the other clusters. We used population structure analysis rather than clustering to examine ancestry. Ancestry distribution of K-3 (red, green, and yellow are indicators of various ancestries) showed all ancestries are present in Africa (Figure 5), whereas ancestry analysis suggested that large numbers of the watermelon cultivars currently available in Africa are not yet used in breeding programs in the rest of the world. Ancestry colored yellow was more predominant in Asia and Europe. North and South American accessions were predominantly red. Population structure analysis provided strong evidence for multiple parallel domestications across Africa.

### Characterization of genome-wide LD

**Figure 6:**
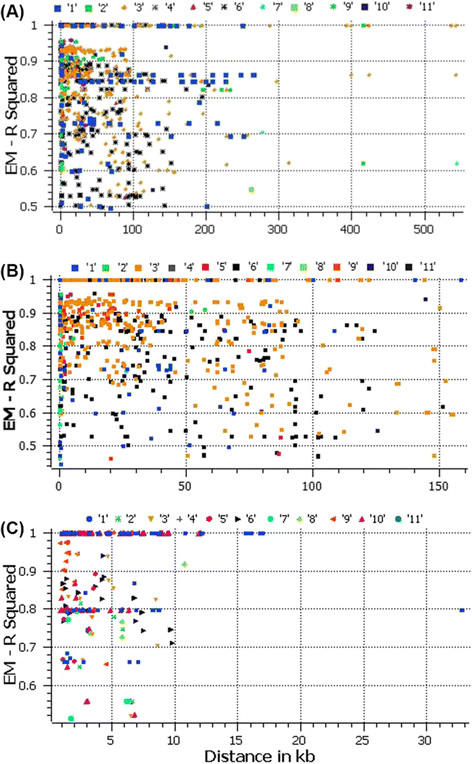
**Genome-wide linkage disequilibrium (LD) across various watermelon chromosomes when compared using A) individual SNPs, B) SNP haplotypes, and C) within individual genes.**

We conducted an extensive LD analysis on the entire dataset of 183 sweet watermelons, on all adjacent marker pairs within a chromosome or within a haplotype block. The results provided values for both the expectation-maximization (EM) algorithm and composite haplotype method (CHM). R^2^ (squared-allele frequency correlations) and D' (LD estimate) values for the EM and CHM methods are given in Additional file 2: Table S1, Additional file 3: Table S2, and Additional file 4: Table S3. We created LD plots using marker-pair associations of adjacent SNPs within a chromosome, adjacent SNPs within a haplotype block, and adjacent SNPs within genes (Figure 6A, B, and C).

**Figure 7:**
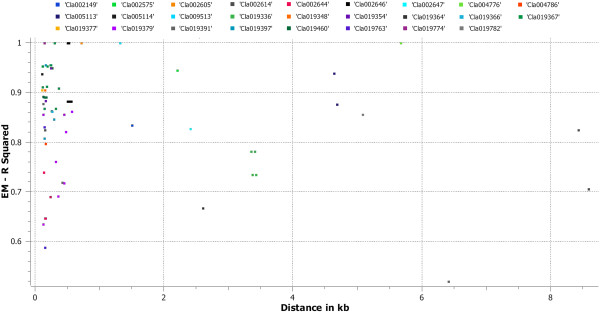
**LD distribution within 26 genes on chromosome 3.**

**Figure 8:**
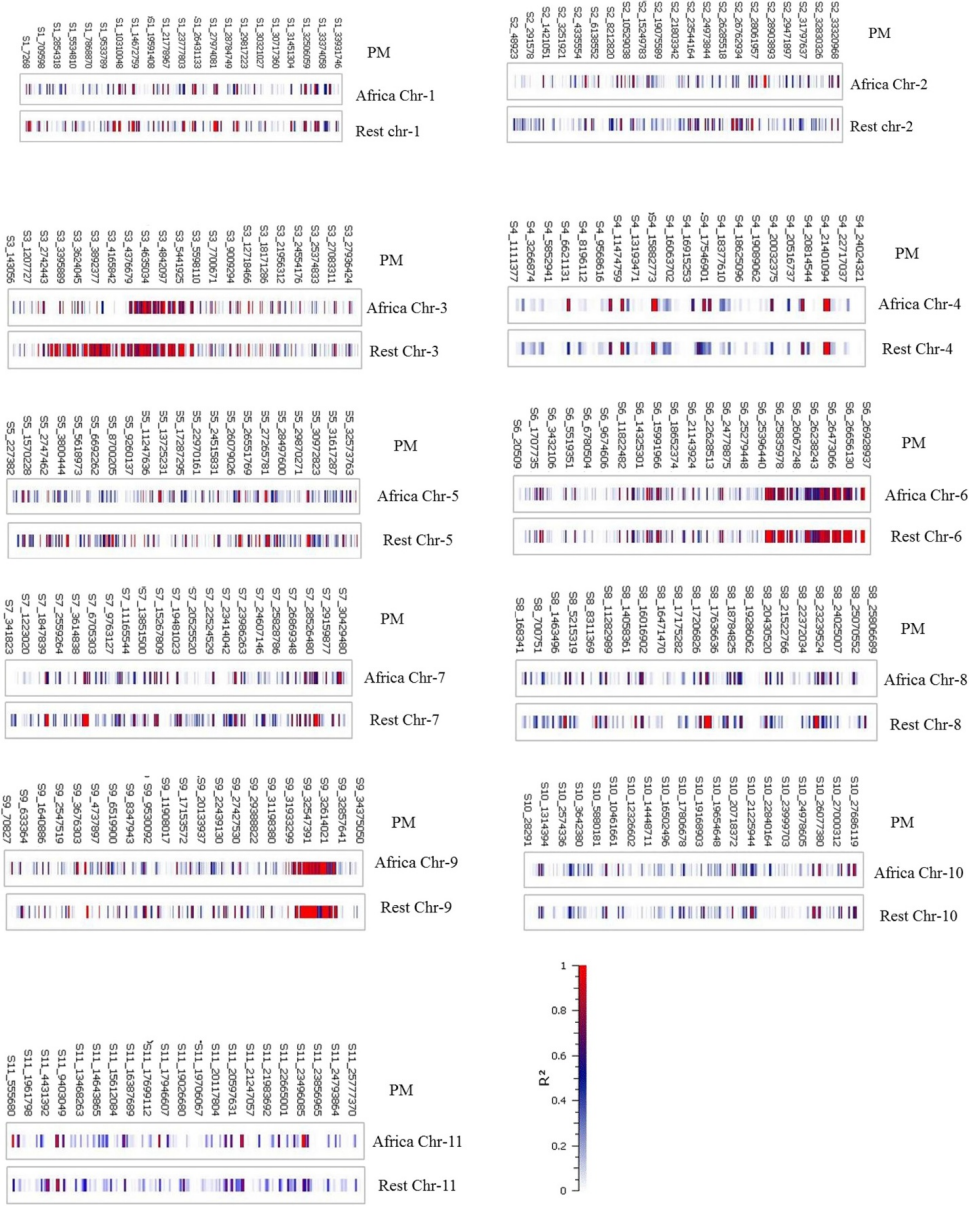
**Comparison of LD distribution across various chromosomes between watermelon accessions from Africa and the rest of the world.**

LD decay varied along chromosomes, with regions of high LD interspersed with regions of low. Pairwise LD was estimated by r^2^ and we compared the pattern of decay at different levels. First, when performing pair-wise analysis considering SNPs across chromosomes, we noted LD decay on average, with an average block size of 100 Kb (Figure 6A). Second, analysis based on adjacent SNPs within haplotypes revealed LD decay within 50 Kb (Figure 6B). Third, analysis of the SNPs located in exons revealed LD decay within 5 Kb (Figures 6C, and Additional file 5: Figure S2, Additional file 6: Figure S3, Additional file 7: Figure S4, Additional file 8: Figure S5, Additional file 9: Figure S6, Additional file 10: Figure S7, Additional file 11: Figure S8, Additional file 12: Figure S9, Additional file 13: Figure S10 and Additional file 14: Figure S11). On chromosome 3, 26 genes under LD were identified (Figure 7). Chromosome 3 appeared to harbor a large extent of LD, followed by chromosomes 6 and 9. When analyzed separately and including various accessions from Africa and the rest of the world, lower LD was noted for all chromosomes in African accessions as compared with those from the rest of the world. Narrow genetic diversity increased LD significantly (Figure 8). On chromosome 3, the LD covered a block of 2.85 Mb in cultivars from the rest of the world, which indicates strong selection in the region, but covered 1.2 Mb in African accessions. The expansion of this LD block in cultivars from the rest of the world appears to be a hitchhiking effect rather than a selective sweep because of the narrow genetic diversity. We identified 257 haplotypes with 769 SNPs (Additional file 15: Table S4). A list of the genes across various chromosomes and the extent of LD within genes are given in Additional file 2: Table S1.

### Selective sweeps and domestication signature characterization

**Figure 9:**
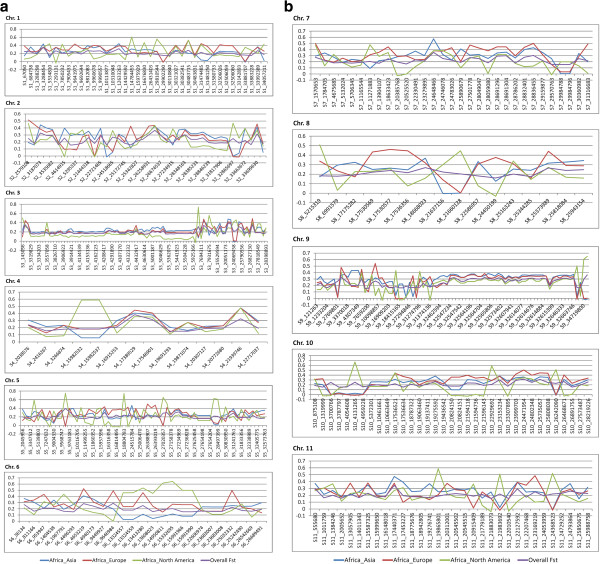
**Genome-wide window based scans of pairwise** ***F***_***ST***_ **for accessions from Asia, Europe, and the Americas compared with those from Africa.** Selection signatures can be seen on parts of chromosome 3 and 9, where *F*_*ST*_ distribution revealed distinct sweeps.

Highly significant pairwise *F*_*ST*_ (P < 0.001) distribution in sweet watermelon accessions from different geographical areas is illustrated in Figure 9a and b. The African and American groups showed high genetic diversity relative to Asian and European groups. Furthermore, the patterns of *F*_*ST*_ variation indicated genomic areas with selective sweep signatures and patterns of world watermelon breeding practices. Selection signatures detected loci with large effects under strong selection on chromosomes 3 and 9.

By scanning the chromosome 3 genome at the selective sweep location, especially in the 1.2 Mb LD block, we identified potential gene candidates selected during sweet watermelon domestication. We identified 50 candidate genes within 1.2 Mb of the genome; Therefore, this region is the most significant for domestication (Additional file 16: Table S5) with important roles in ripening, sugar-mediated signaling and carbohydrate transport, fruit development, nitrate transmembrane transporter, cytochrome P450, pectinesterase/pectinesterase inhibitor, zinc finger (CCCH-type) family protein, glyceraldehyde-3-phosphate dehydrogenase, pectate lyase family protein, and catalytic/cation binding/hydrolase.

### Implementation of a medium-resolution GWAS for the fresh juice SSC trait

**Figure 10:**
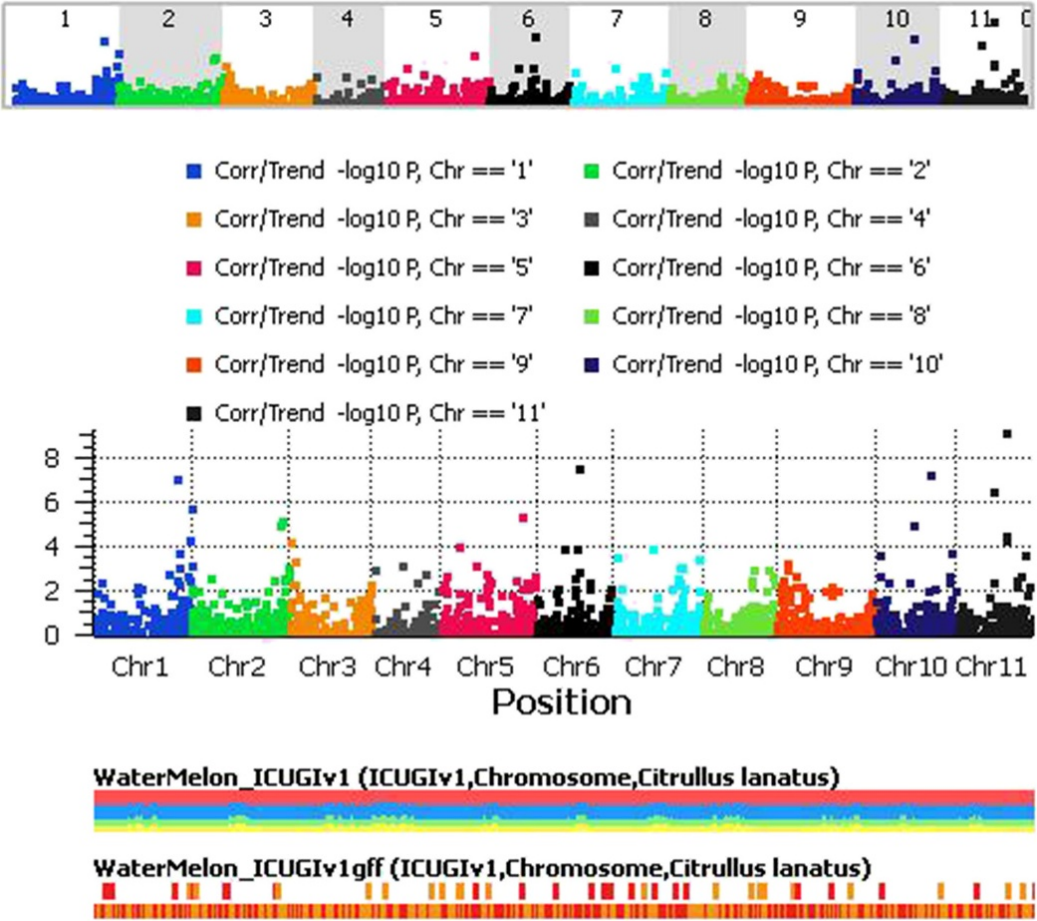
**Manhattan plot of the genome-wide association study for the soluble solids trait.** Chromosome coordinates are displayed along the X-axis with the negative log 10 of the association P-value for each single nucleotide polymorphism displayed on the Y-axis. A higher negative log 10 indicates stronger association with the trait.

A set of 96 genotypes were grown in controlled conditions and the means for the SSC trait clearly followed a normal distribution. Therefore, the trait is under the control of multiple genes (Additional file 17: Figure S12). We used a GWAS with 5,254 SNPs to identify alleles that affect total SSC. Results pertaining to the GWAS are presented in a Manhattan plot (Figure 10). In Manhattan plots, genomic coordinates are displayed along the X-axis with the negative log 10 of the association P-value for each single nucleotide polymorphism on the Y-axis. Because the strongest associations have the smallest P-values, their negative logarithms will be the greatest. In this study, four SNPs were associated with total SSC after Bonferroni correction according to the EMMAX model, which corrects for population structure as well as identity by descent (IBD). The marker S1_28788452 (Bonferroni P = 0.0003) is located on chromosome 1. This SNP is a synonymous mutation for leucine and is located in the exon of the gene Cla014168, a ubiquitin-protein ligase with R = 0.54. Allele A was the minor allele with a frequency of 0.07 and 100% call rate. S6_15135822 is a non-synonymous mutation causing a Gln → Lys change on Cla002989, an unknown gene. This marker was associated with a Bonferroni P = 0.0001 and a minor allele frequency (allele A) of 0.1, with a call rate of 97%. The strength of association was negative (R^2^=0.57). Two other SNPs (S11_17440371 and S10_19206736) were positively associated with SSC, with R^2^ = 0.63 and 0.57, and could withstand Bonferroni correction (2.36E-06 and 0.0002, respectively). The MAFs for these two SNPs (A and G) were 0.18 and 0.05, with call rates of 99% and 94%, respectively. S11_17440371 is located in the intergenic region of Cla023099 and Cla023100, which code for Profilin and PPR repeat protein, respectively. S10_19206736 is located in the Cla017168 promoter region, its function is unknown.

## Discussion

### Genotyping by sequencing

Many of the challenges posed by complex crop genomes can be overcome by GBS [13, 30]. This protocol is a multiplexed, high-throughput, low-cost method to explore genetic diversity in populations [11]. In this paper, we report a robust set of 11,485 SNPs mapped to various chromosomes with a MAF of ≥ 0.01. Sandlin et al. [31], Ren et al. [32], and Nimmakayala et al. [5] developed 1,073, 386, and 384 SNPs, respectively, for genetic mapping and diversity studies in watermelon. Guo et al. [33] re-sequenced 20 watermelon accessions including sweet, semi-wild, and wild watermelons to identify 6,784,860 candidate SNPs and 965,006 small insertions/deletions (indels). We used the largest set of a cultivated collection of 183 accessions representing all of the important ecotypes from around the globe to resolve the diversity of cultivated watermelon, which will allow for incorporating diverse alleles into cultivated watermelons worldwide.

### Domestication and divergence of sweet watermelon

We identified three sweet watermelons clusters within Africa and related one of those to the sweet watermelon accessions from the rest of the world. The other two African sweet watermelon clusters were not used in watermelon breeding programs in other parts of the world. Therefore, the underutilized accessions from these clusters could be an important resource for widening watermelon cultivar genetic diversity. Founder effects based on relatively few cultivars appear to account for the prevailing narrow genetic diversity in global cultivar germplasm. This finding agrees with previous results [9, 33], in particular, the lower diversity among American, Chinese, and East Asian types.

### Location of selective sweeps across the genome

Domestication effects lead to complete fixation of the genomic regions that harbor alleles of importance by purifying selection. The loss of nucleotide diversity in the flanking regions [26, 34] is known as the hitchhiking effect and a region of the genome in which selection has driven a haplotype to complete fixation is defined as having undergone a selective sweep [26, 35, 36]. Such regions may also occur within the genome because of random drift and are not distinguishable from regions that have undergone a selective sweep. We minimized the errors in locating the regions that have undergone selective sweeps caused by random drift or narrow genetic diversity by including a large diverse panel of accessions derived from different ancestral populations, e.g., Asia, Europe, and America as well as a wide range of ancestral populations from Africa. The selective sweep approach is a type of reverse genetic tool that begins with a selection signature and attempts to infer the selected mutation and its associated phenotype [37]. In contrast, a GWAS is a forward genetics approach that progresses from a phenotype to the identification of underlying causal genes and mutations. The SNPs discovered by GBS allowed to compare the pairwise *F*_*ST*_ for accessions from Asia, Europe, and the Americas to those from Africa. By scanning a moving window of these pair-wise *F*_*ST*_ values across all chromosomes, we could identify selective sweep regions to assess the effects of selection during the breeding history of cultivated watermelon. The selection of individuals with favorable mutations during domestication and by breeding practices has led to reduced genetic diversity in crop species [38]. Pairwise *F*_*ST*_ distributions on numerous chromosomes reflected the breeding history patterns across several continents.

LD is a key factor in determining the number of markers needed for a GWAS and genomic selection (GS). Using genome-wide SNPs, we evaluated the genome-wide LD pattern for a diverse collection of sweet watermelon. Genomes with high LD will require low marker density for GWASs or GS, therefore our SNPs may be of immense use for GWASs of watermelon breeding. In the current research, we characterized extensive LD in the watermelon collections of Asia, Europe, and the Americas as compared with those of Africa, possibly because of less divergent cultivar pools. GWASs can be implemented with moderate marker density in barley and alfalfa because these populations contain extensive admixture LD, as does watermelon [27, 39]. By selecting subsets of genotypes from globally cultivated watermelons, breeders can modulate LD to acceptable levels and build suitable association mapping panels for genome-wide marker-based breeding projects. A complete understanding of LD within genes is important for realizing the impact of marker-assisted selection. If a particular gene is under high LD, marker-assisted selection can be efficiently performed with a single marker irrespective of its location in or near the gene. In contrast, if a gene is under low LD, several markers at various positions of the gene must be used to transfer a complete allele because recombination can occur within the gene. In our study, we characterized genic LD across all of the chromosomes.

### Usefulness of the current diversity panel for future association mapping studies

GWASs based on LD provide a promising tool for detecting and fine-mapping quantitative trait loci underlying complex traits. In this study, we explored the genetic basis of variation for the total SSC trait in a diverse watermelon collection of global origin. Despite the advantages of GWAS in pinpointing genetic polymorphisms underlying traits, this approach may incur inflated false-positive findings because of population structure [28, 40, 41]. Imprecise GWAS modeling would result in spurious marker associations if the model cannot precisely correct for population stratification [42, 43]. Variance component approaches, such as efficient mixed-model association (EMMA), can correct for a wide range of sample structures by explicitly accounting for pairwise relatedness between individuals, using high-density SNP markers to model the phenotype distribution [44]. In this study, we used a more efficient EMMA, eXpedited (EMMAX), which reduces the computational time for analyzing large GWAS data sets and includes PCA eigen vectors and identity by descent (IBD) matrices in correcting for sample structure [42, 45].

## Conclusions

Analysis of genetic diversity in world collections of cultivar accessions helps understand LD decay at various levels. Because LD in the watermelon is quite high, the marker set we developed would be sufficient for a low-power GWAS of sweet watermelon cultivars; a high-power study might require up to 50,000 SNPs with a MAF = 0.05. From this pilot study, GBS can effectively detect genome-wide SNPs and provide a powerful tool for the systematic exploration of global watermelon collections. Re-sequencing strategies to develop millions of SNPs for crops such as maize and rice [46–49] have shown what is possible for the watermelon research community [5]. The identification of watermelon SNPs, as in this and previous studies, will allow for genome-wide association mapping and marker-assisted selection to support breeding programs.

## Methods

We used 183 accessions of *C. lanatus* var. *lanatus* representing sweet watermelon from a wide geographical area of the world (Additional file 18: Table S6). We grew a random selection of 96 accessions, five plants per accession, in three replications under controlled growth conditions and standard management practices. Five fruits at the ripening stage per replication were harvested by standard horticultural procedures. Data for the SSC trait in flesh juice (i.e., Brix%) for five fruits from three plants were recorded with a hand refractometer (ATAGO, Japan).

### SNP discovery by GBS

Genomic DNA was isolated with a DNeasy® Plant Mini Kit (QIAGEN, Germany). GBS was performed as described by [11] at the Institute of Genomic Diversity (Cornell University). Briefly, genome complexity was reduced by digesting total genomic DNA from individual samples with the *Ape*KI methylation sensitive restriction enzyme. Digested products were then ligated to adapter pairs with enzyme-compatible overhangs; one adapter contained the barcode sequence and a binding site Illumina sequencing primer. The samples were then pooled, purified, and amplified with primers compatible to the adapter sequences. The PCR primers also added 3′ sequences complementary to the solid-phase oligonucleotides that coat the Illumina sequencing flow-cell. After a short PCR cycle, the pooled products were purified; GBS library fragment-size distributions were checked on a BioAnalyzer (Agilent Technologies, Inc., USA). The PCR products were quantified and diluted for sequencing on the Illumina HiSeq 2500 (Illumina Inc., USA). Chromosomal assignment and position on the physical map of candidate genes, GBS markers, were deduced from the watermelon Whole Genome Sequence (WGS) draft at http://www.icugi.org.

### Genetic diversity and population structure analysis

For quantitative assessment of the number of clusters in the association mapping panel, we used a Bayesian clustering analysis with a model-based approach implemented in STRUCTURE v2.2 [50]. This approach involves use of multi-locus genotypic data to assign individuals to clusters or groups (k) without prior knowledge of their population affinities. The program was run with SNP markers for k-values 1–9 (hypothetical number of subgroups), with 100,000 burn-in iterations, followed by 500,000 Markov Chain Monte Carlo (MCMC) iterations for accurate parameter estimates with a high-performance cluster. To verify the consistency of the results, we performed three independent runs for each K. An admixture model with correlated allele frequencies was used. The optimal K value was determined by use of an ad-hoc statistic, ΔK [51]. The number of *K*s in each dataset was evaluated by ΔK values estimated with the software Structure Harvester, a website (http://www.taylor0.biology.ucla.edu/structureHarvester) and program for visualizing STRUCTURE output and implementing the Evanno method. In a second approach, we used PCA with the SNP & Variation Suite (SVS v8.1.5) (Golden Helix, Inc., Bozeman, MT, USA; http://www.goldenhelix.com).

### Analysis of selective sweeps

*F*_*ST*_ estimation was based on Wright’s F statistic [52] and deviation from HWE in SVS v8.1.5. The significance of differences between *F*_*ST*_ values was assessed in exact tests. Annotation and gene ontology terms for genes from the selective sweeps were identified using the WGS draft for watermelon [33].

### Mapping with GWAS

For GBS data, we only considered the SNPs successfully mapped to the watermelon WGS draft, because knowing the chromosome location of SNPs helps prevent spurious LD and thereby unreliable association mapping. Mapped SNPs were further filtered by call rate >90% and HWE = 0.001. Before studying LD decay, haplotype blocks were calculated for all markers using the default settings in SVS v8.1.5. Adjacent and pairwise measurements of LD for GBS data were calculated separately for SNPs in each individual chromosome. All LD plots as well as LD measurements and haplotype frequency calculations were carried out in SVS v8.1.5 and Tassel 5.0 (http://www.maizegenetics.net). For GWAS, the population structure Q matrix was replaced by the principal component matrix [53]. The P matrix (PCA matrix) and identity by descent (IBD) was calculated from LD-pruned SNPs in SVS v8.1.5. GWAS involved a single-locus mixed linear model developed by the EMMAX method [42] and implemented in SVS v8.1.5. We used a PCA matrix (first two vectors) and the IBD matrix to correct for population stratification. Manhattan plots for associated SNPs were visualized in GenomeBrowse v1.0 (Golden Helix, Inc). The SNP P-values from GWAS underwent sequential Bonferroni correction [54] as well as false discovery rate (FDR) analysis [55].

### Availability of supporting data

All of the supporting data are included as additional files.

## Electronic supplementary material

Additional file 1: Figure S1: Delta K distribution across various clusters as estimated using Structure Harvester. K3 showed the highest peak indicating that three clusters sufficiently define watermelon population structure. (TIFF 622 KB)

Additional file 2: Table S1: List of significant marker associations and respective LD values estimated for pair-wise adjacent SNPs within chromosomes. (XLSX 127 KB)

Additional file 3: Table S2: List of significant marker associations and respective LD values estimated for pair-wise adjacent SNPs within haplotypes. (XLSX 46 KB)

Additional file 4: Table S3: List of significant marker associations and respective LD values estimated for pair-wise SNPs within genes located on various chromosomes. (XLSX 163 KB)

Additional file 5: Figure S2: LD distribution within candidate genes on chromosome 1. (JPEG 539 KB)

Additional file 6: Figure S3: LD distribution within candidate genes on chromosome 2. (JPEG 475 KB)

Additional file 7: Figure S4: LD distribution within candidate genes on chromosome 4. (JPEG 377 KB)

Additional file 8: Figure S5: LD distribution within candidate genes on chromosome 5. (JPEG 538 KB)

Additional file 9: Figure S6: LD distribution within candidate genes on chromosome 6. (JPEG 502 KB)

Additional file 10: Figure S7: LD distribution within candidate genes on chromosome 7. (JPEG 522 KB)

Additional file 11: Figure S8: LD distribution within candidate genes on chromosome 8. (JPEG 529 KB)

Additional file 12: Figure S9: LD distribution within candidate genes on chromosome 9. (JPEG 549 KB)

Additional file 13: Figure S10: LD distribution within candidate genes on chromosome 10. (JPEG 510 KB)

Additional file 14: Figure S11: LD distribution within candidate genes on chromosome 11. (JPEG 486 KB)

Additional file 15: Table S4: List of SNP haplotypes across the watermelon genome. (XLS 56 KB)

Additional file 16: Table S5: List of candidate genes that harbor important mutations for domestication identified from the selective sweep on chromosome 3 region. (DOCX 23 KB)

Additional file 17: Figure S12: Normal distribution pattern of total soluble solids means measured across various accessions. (JPEG 34 KB)

Additional file 18: Table S6: List of accessions used in the current study. (XLSX 20 KB)

## Abbreviations

EMMA: Efficient mixed-model association
EMMAX: Efficient mixed-model association eXpedited
FDR: False discovery rate
*F*_*ST*_: Fixation index
GBS: Genotyping by sequencing
GWAS: Genome-wide association study
HWE: Hardy–Weinberg equilibrium
IBD: Identity by descent
LD: Linkage disequilibrium
MAF: Minor allele frequency
PCA: Principal component analysis
MLM: Mixed linear model.
