# Supplementary figures and images for: Single nucleotide polymorphisms generated by genotyping by sequencing to characterize genome-wide diversity, linkage disequilibrium, and selective sweeps in cultivated watermelon
Source: BMC Genomics. 2014 Sep 8;15(1):767. doi: 10.1186/1471-2164-15-767 (PMC4246513; doi:10.1186/1471-2164-15-767)

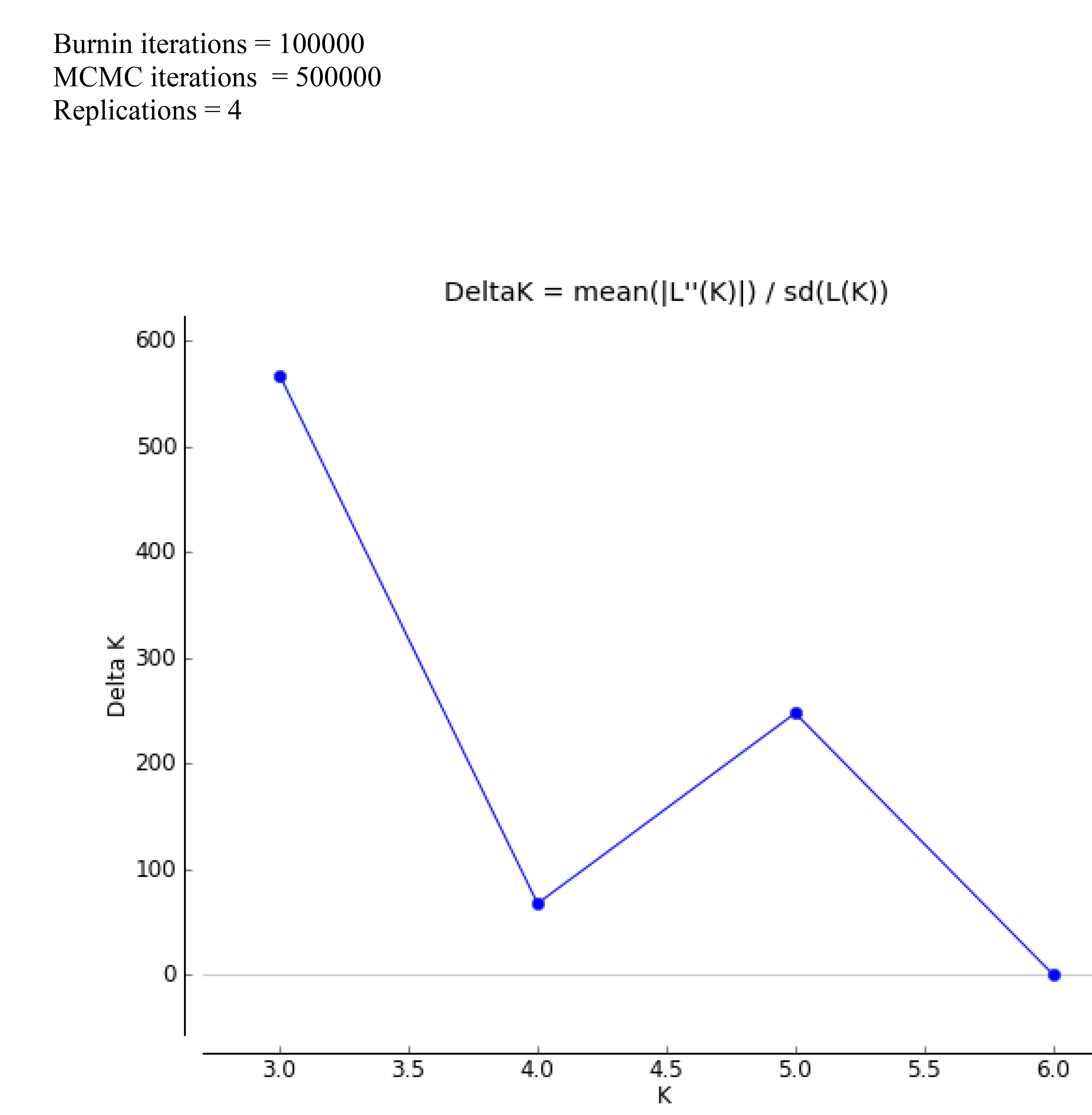

Supplement: Supplementary file 1 — Additional file 1: Figure S1: Delta K distribution across various clusters as estimated using Structure Harvester. K3 showed the highest peak indicating that three clusters sufficiently define watermelon population structure. (TIFF 622 KB) [file 12864_2014_6684_MOESM1_ESM.tiff]

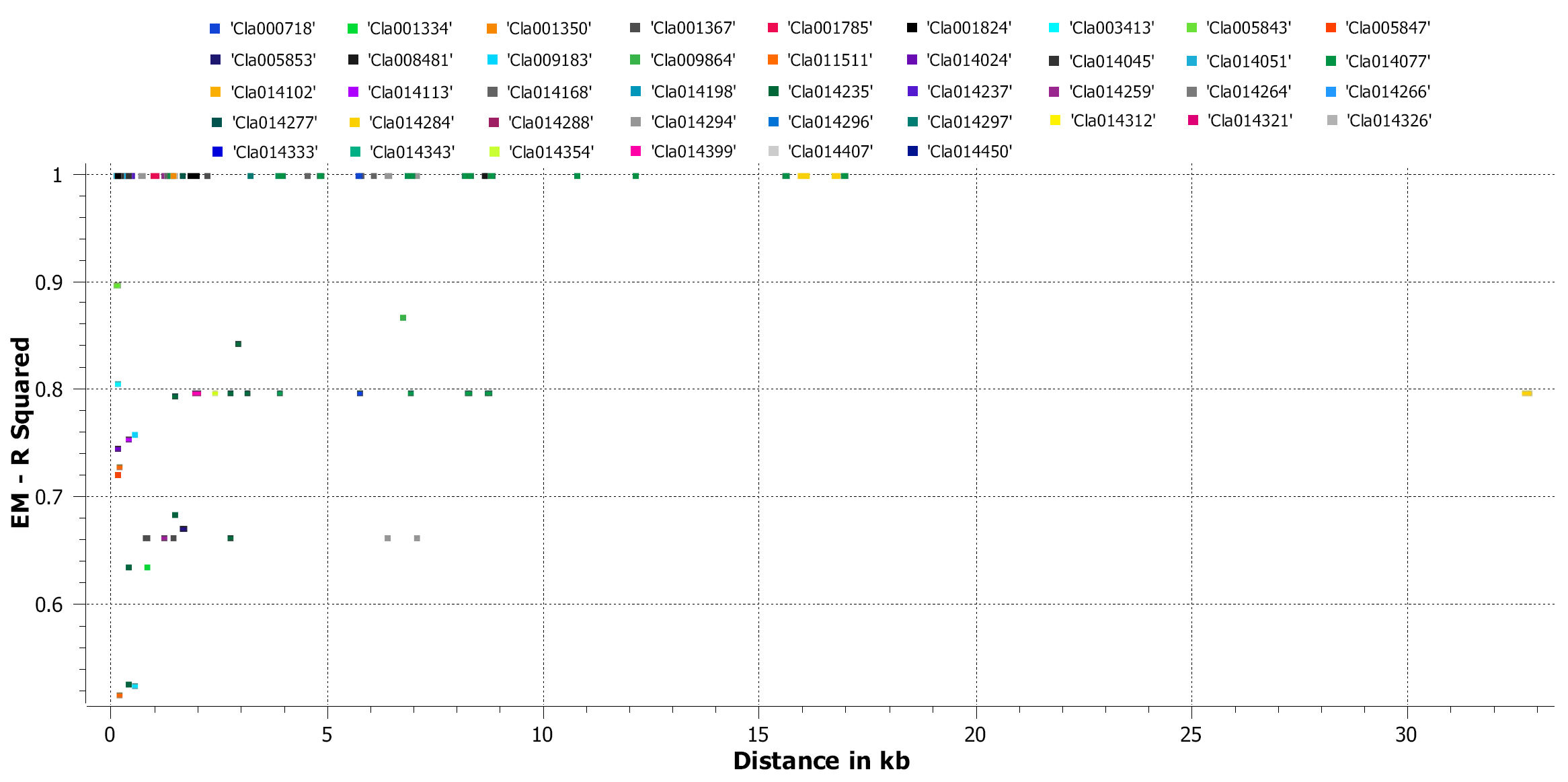

Supplement: Supplementary file 5 — Additional file 5: Figure S2: LD distribution within candidate genes on chromosome 1. (JPEG 539 KB) [file 12864_2014_6684_MOESM5_ESM.jpeg]

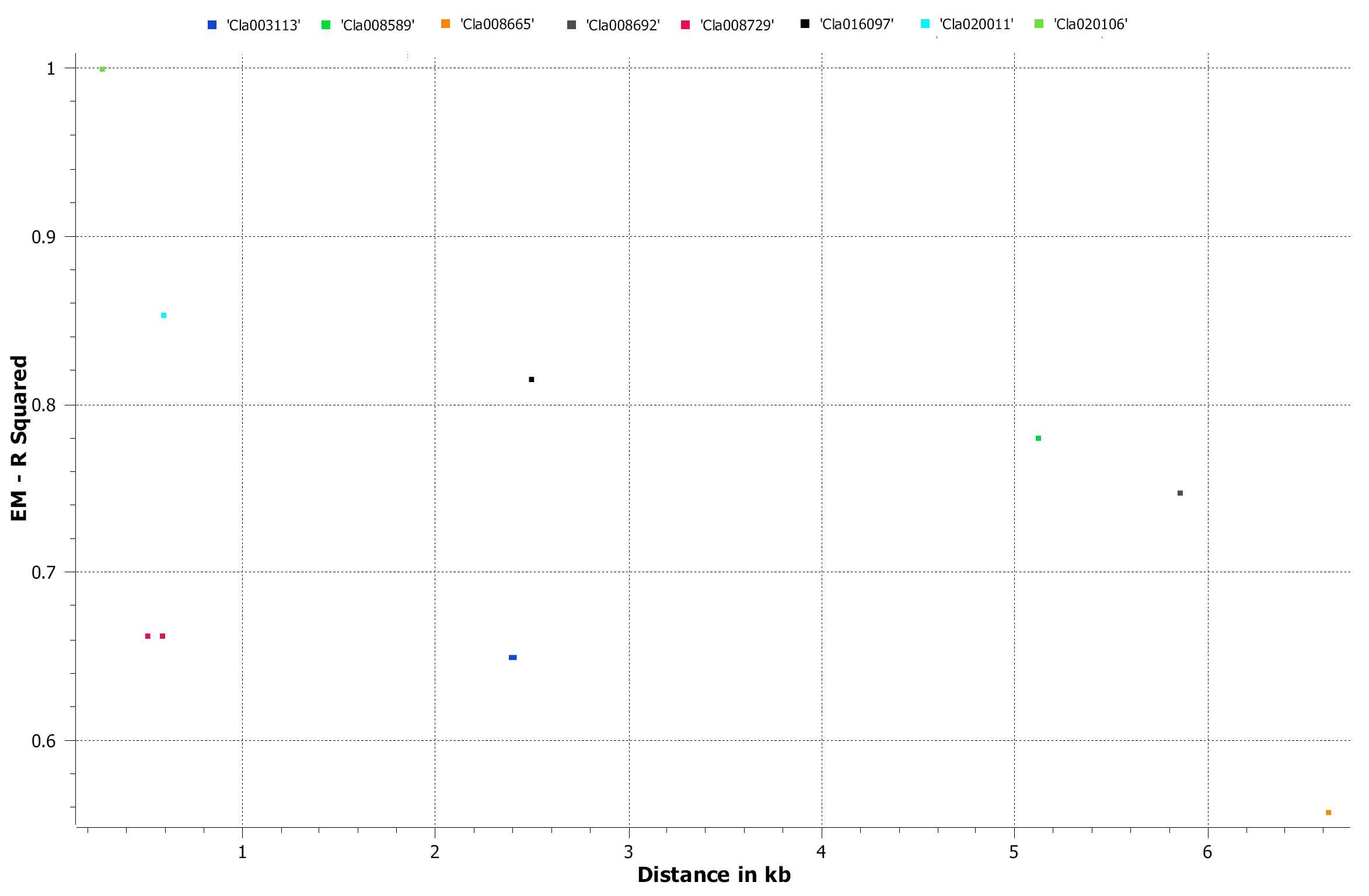

Supplement: Supplementary file 6 — Additional file 6: Figure S3: LD distribution within candidate genes on chromosome 2. (JPEG 475 KB) [file 12864_2014_6684_MOESM6_ESM.jpeg]

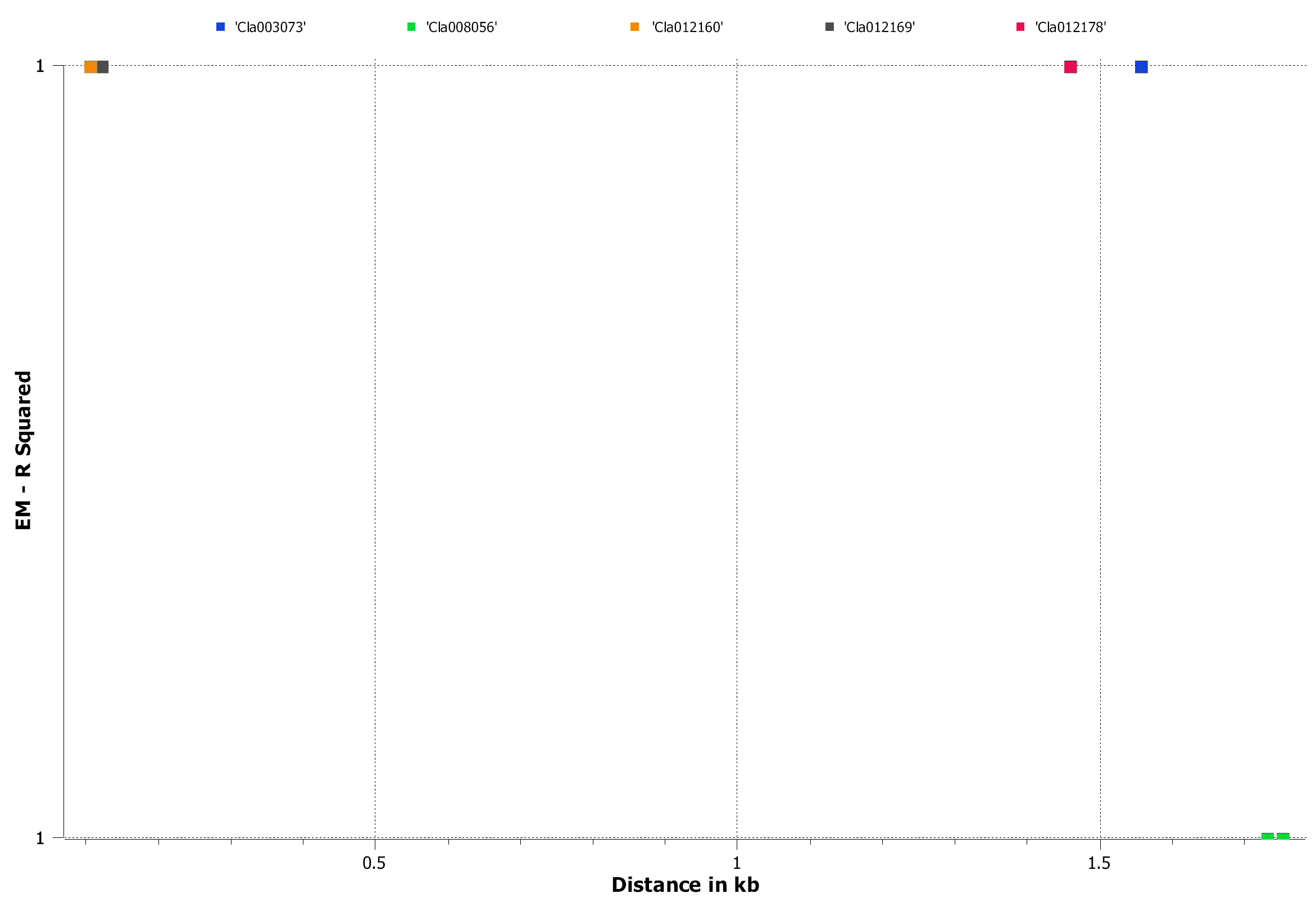

Supplement: Supplementary file 7 — Additional file 7: Figure S4: LD distribution within candidate genes on chromosome 4. (JPEG 377 KB) [file 12864_2014_6684_MOESM7_ESM.jpeg]

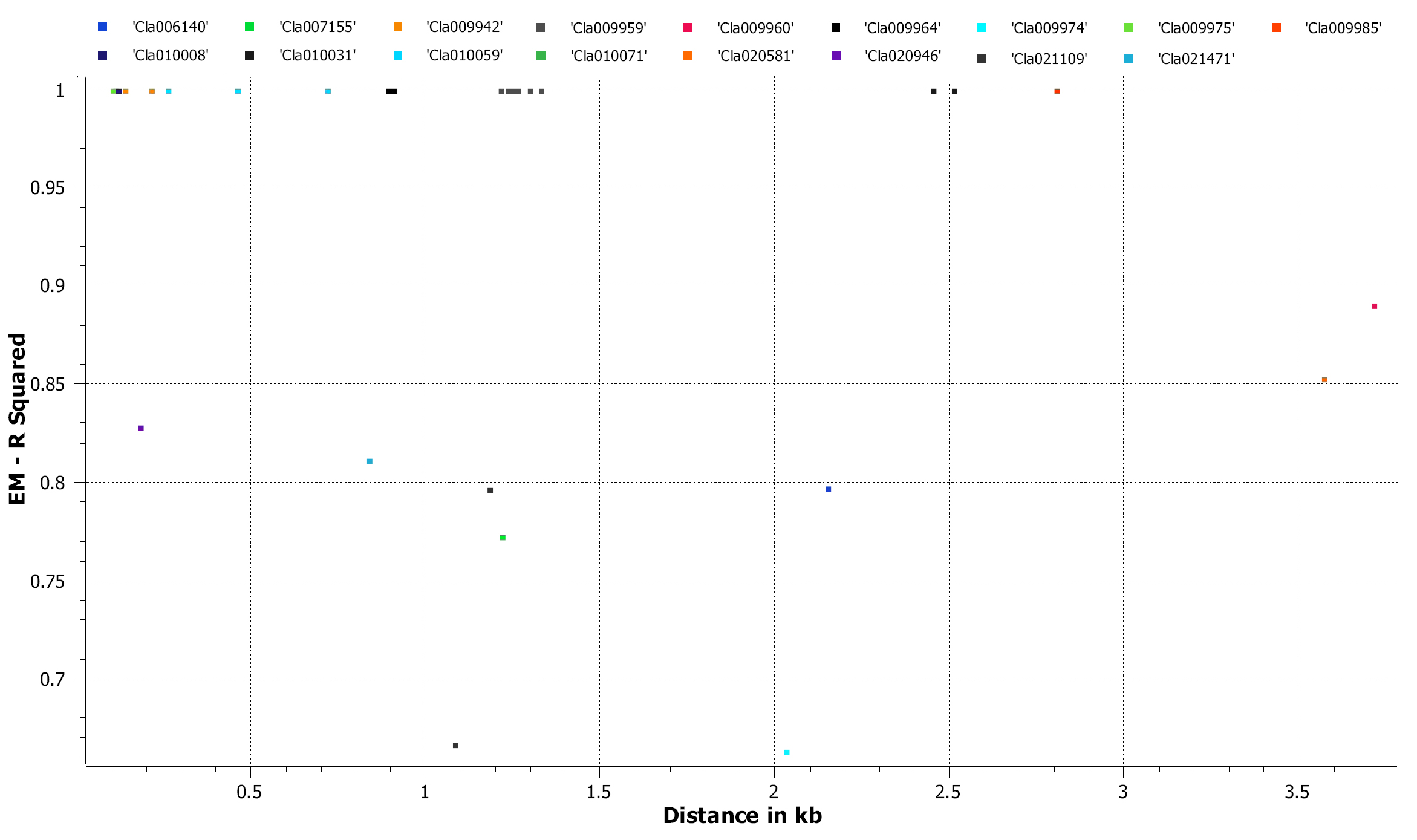

Supplement: Supplementary file 8 — Additional file 8: Figure S5: LD distribution within candidate genes on chromosome 5. (JPEG 538 KB) [file 12864_2014_6684_MOESM8_ESM.jpeg]

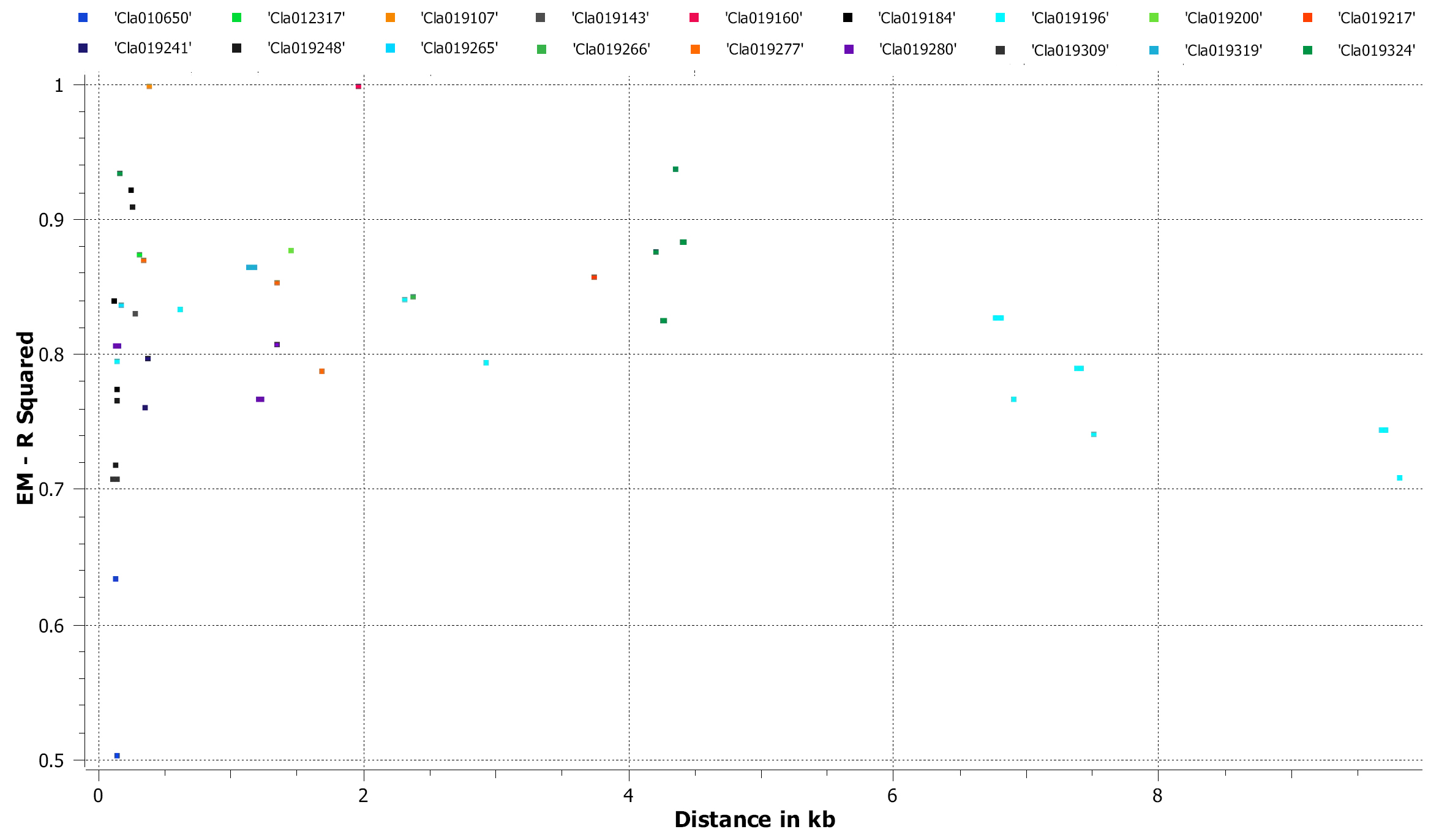

Supplement: Supplementary file 9 — Additional file 9: Figure S6: LD distribution within candidate genes on chromosome 6. (JPEG 502 KB) [file 12864_2014_6684_MOESM9_ESM.jpeg]

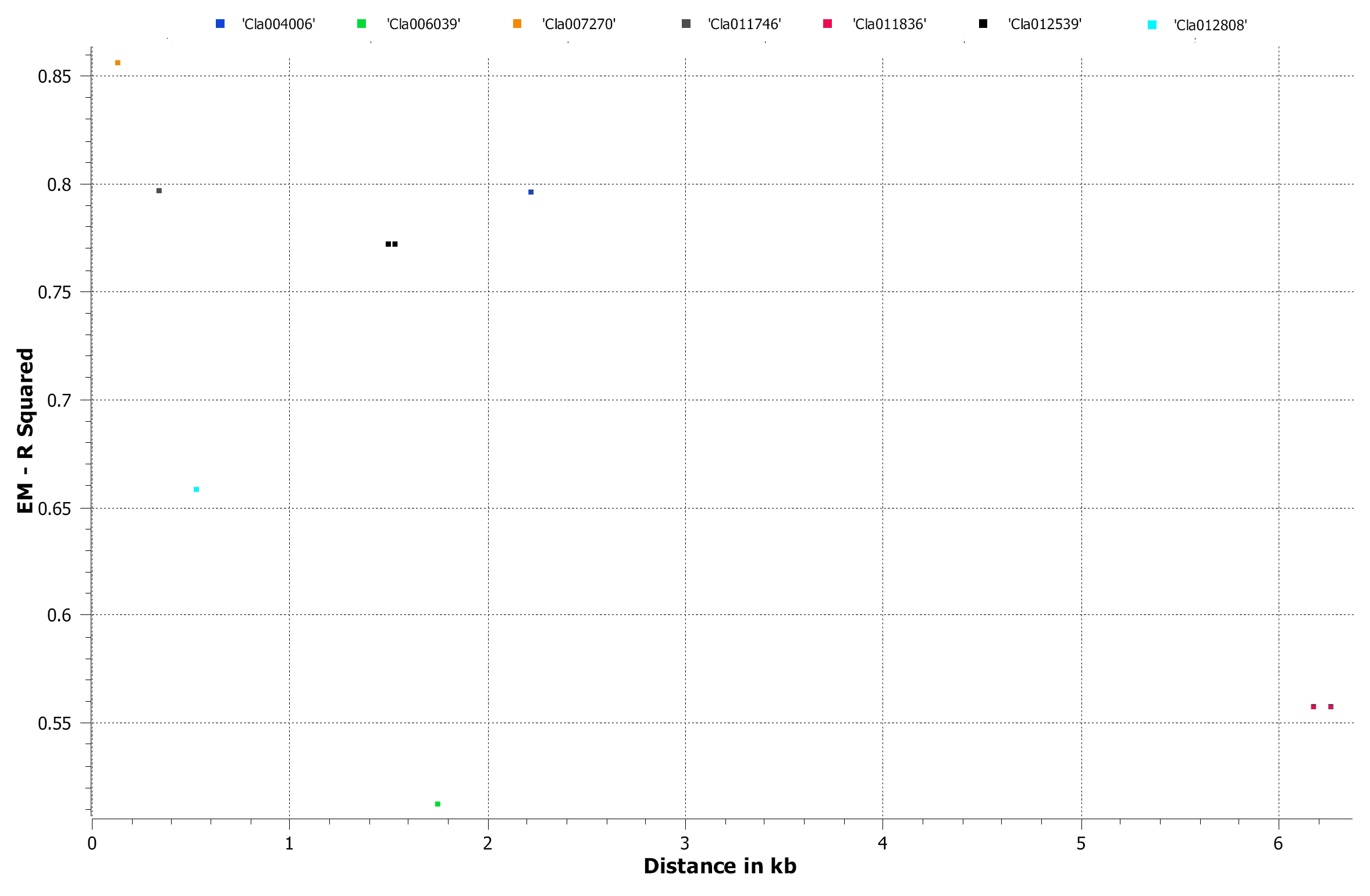

Supplement: Supplementary file 10 — Additional file 10: Figure S7: LD distribution within candidate genes on chromosome 7. (JPEG 522 KB) [file 12864_2014_6684_MOESM10_ESM.jpeg]

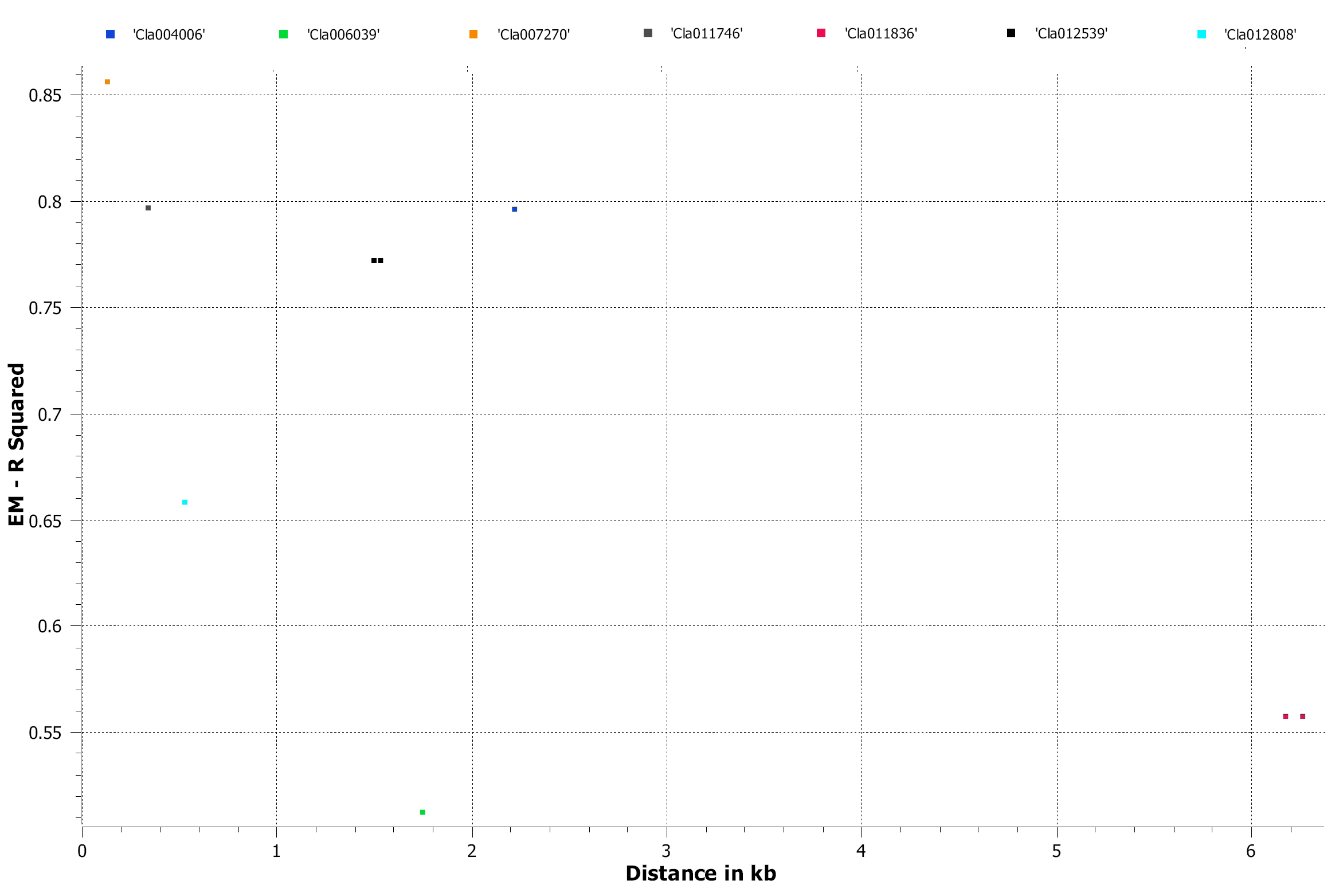

Supplement: Supplementary file 11 — Additional file 11: Figure S8: LD distribution within candidate genes on chromosome 8. (JPEG 529 KB) [file 12864_2014_6684_MOESM11_ESM.jpeg]

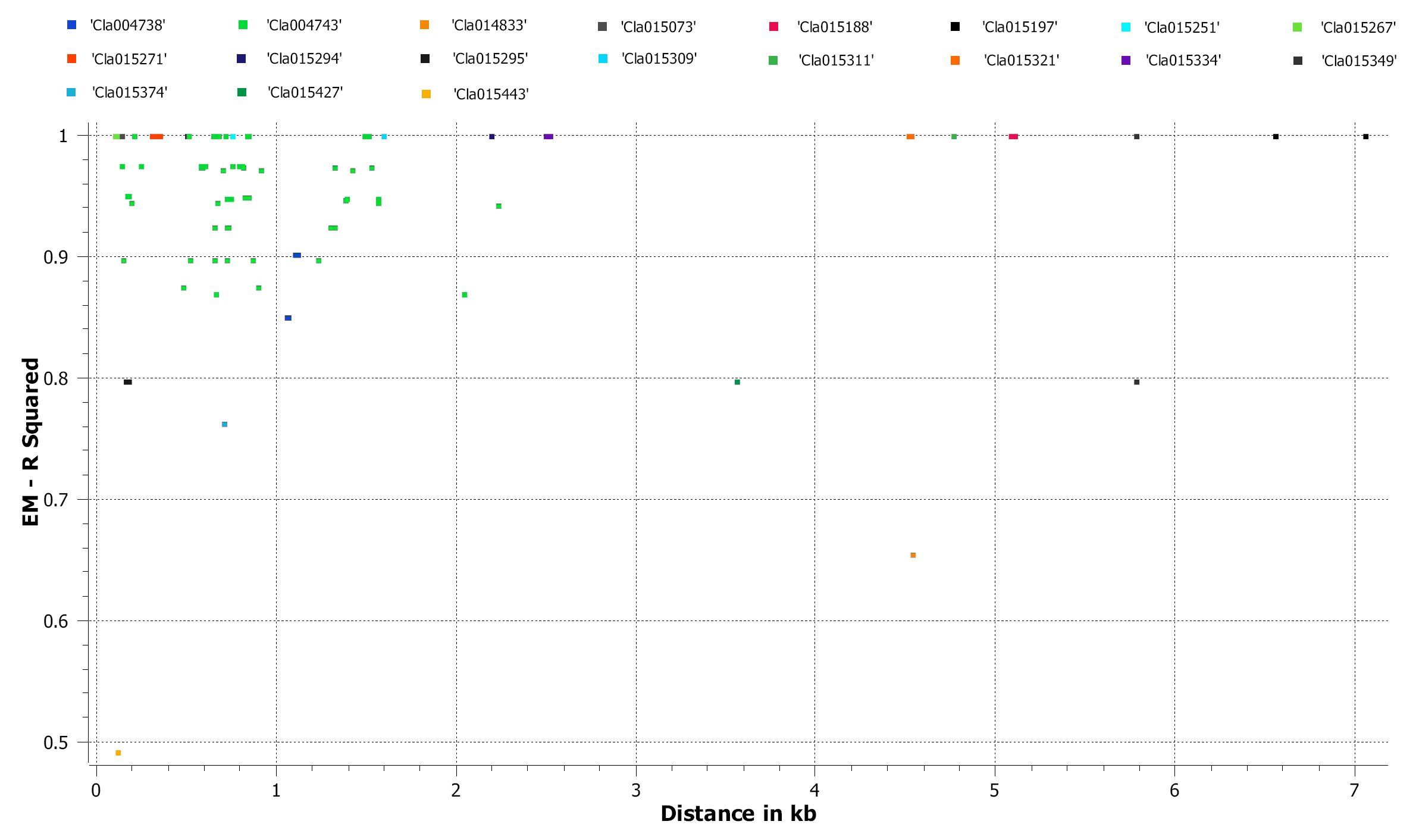

Supplement: Supplementary file 12 — Additional file 12: Figure S9: LD distribution within candidate genes on chromosome 9. (JPEG 549 KB) [file 12864_2014_6684_MOESM12_ESM.jpeg]

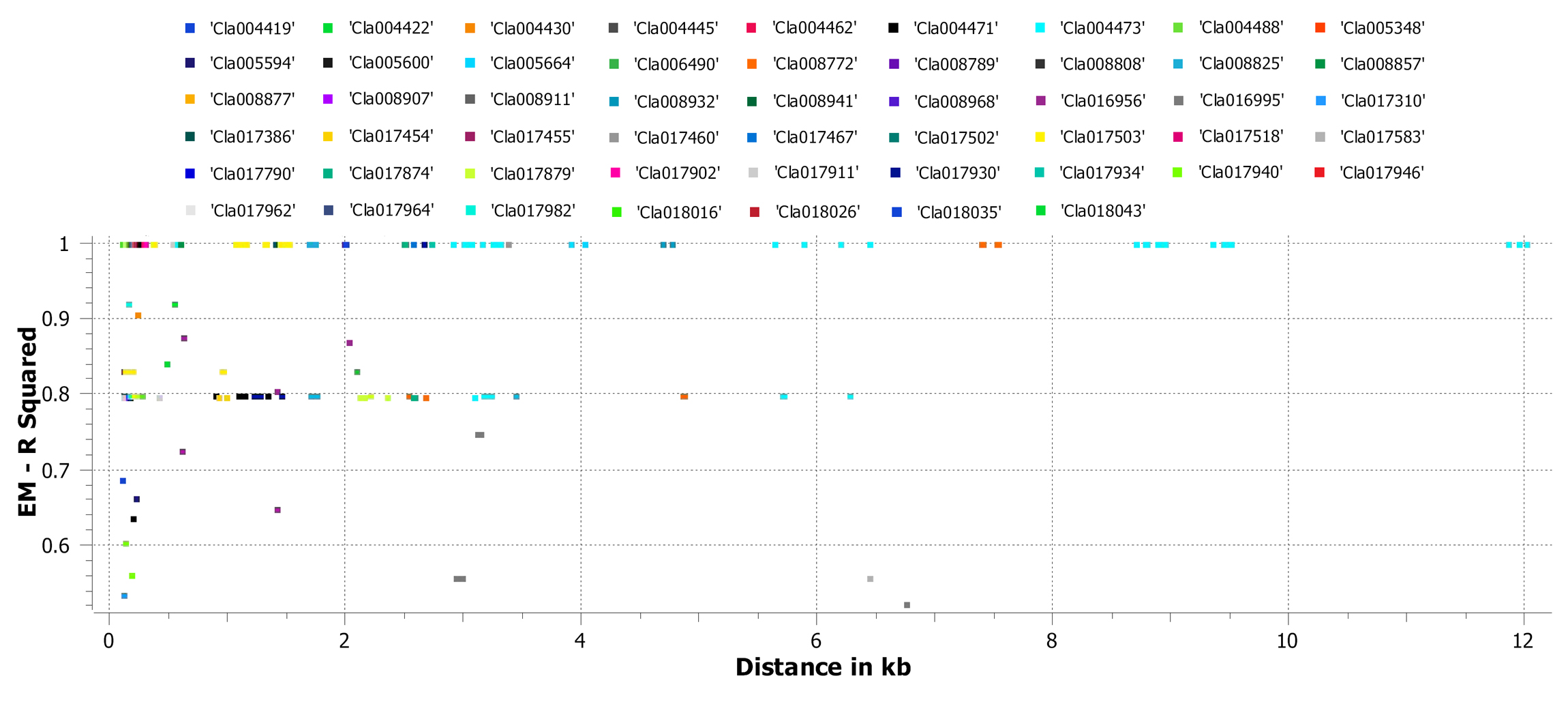

Supplement: Supplementary file 13 — Additional file 13: Figure S10: LD distribution within candidate genes on chromosome 10. (JPEG 510 KB) [file 12864_2014_6684_MOESM13_ESM.jpeg]

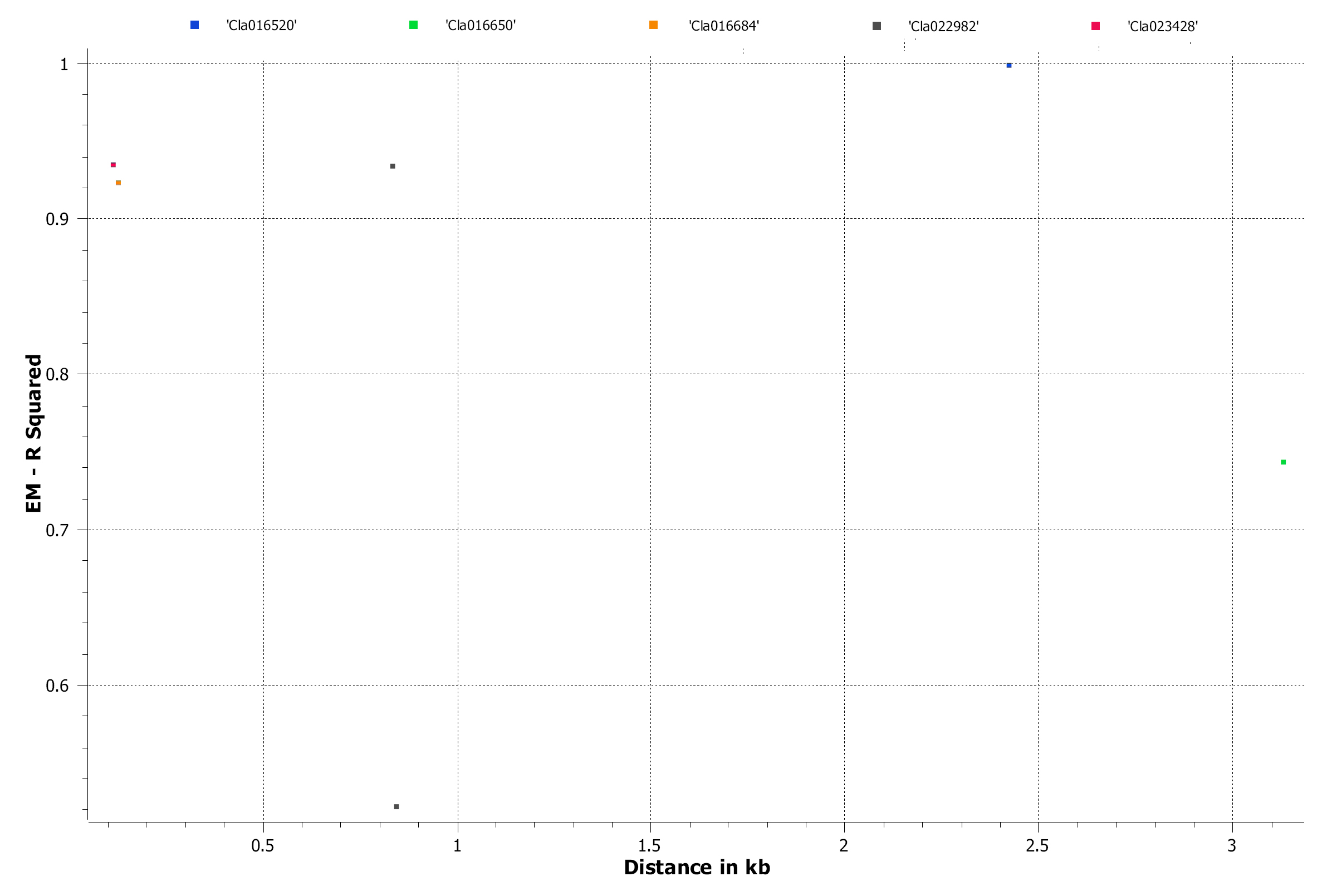

Supplement: Supplementary file 14 — Additional file 14: Figure S11: LD distribution within candidate genes on chromosome 11. (JPEG 486 KB) [file 12864_2014_6684_MOESM14_ESM.jpeg]

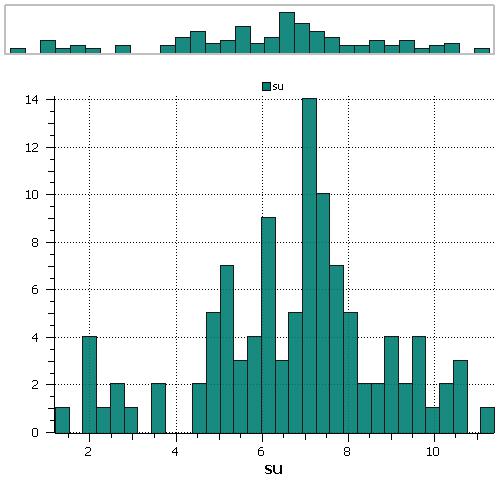

Supplement: Supplementary file 17 — Additional file 17: Figure S12: Normal distribution pattern of total soluble solids means measured across various accessions. (JPEG 34 KB) [file 12864_2014_6684_MOESM17_ESM.jpeg]
