# Supplementary material for: Single nucleotide polymorphisms generated by genotyping by sequencing to characterize genome-wide diversity, linkage disequilibrium, and selective sweeps in cultivated watermelon
Source: BMC Genomics. 2014 Sep 8;15(1):767. doi: 10.1186/1471-2164-15-767 (PMC4246513; doi:10.1186/1471-2164-15-767)
Supplement: Supplementary file 16 — Additional file 16: Table S5: List of candidate genes that harbor important mutations for domestication identified from the selective sweep on chromosome 3 region. (DOCX 23 KB) [file 12864_2014_6684_MOESM16_ESM.docx]

| **Gene ID** | **Position of gene** | **Gene name** | **GO biological process** | **GO Molecular function** | **GO Cellular component** |
| --- | --- | --- | --- | --- | --- |
| Cla005118 | 3392221..3394160 (+) | DEHYDRATION-INDUCED | - | protein binding | intracellular |
| Cla005119 | 3395533..3399166 (-) | serine/threonine-protein kinase WNK3 | protein amino acid phosphorylation | ATP binding | Cytoplasm/membrane/nucleus |
| Cla005121 | 3421045..3421812 (+) | nitrate transmembrane transporter | response to nitrate/wounding | nitrate transmembrane transporter activity | plasma membrane |
| Cla005122 | 3423827..3424849 (-) | Manganese-dependent ADP-ribose/CDP-alcohol diphosphatase | - | metal ion binding | - |
| Cla005123 | 3428968..3433523 (-) | QS (QUINOLINATE SYNTHASE); 4 iron, 4 sulfur cluster binding | NAD biosynthetic process | quinolinate synthetase A activity | Plastid/chloroplast |
| Cla005124 | 3436317..3438709 (-) | Cytochrome P450 | Ripening | oxidoreductase activity/electron carrier activity | integral to membrane/chloroplast |
| Cla005125 | 3438728..3439803 (-) | ” | ” | ” | ” |
| Cla005126 | 3442988..3444434 (-) | ” | ” | ” | ” |
| Cla005127 | 3461211..3463467 (-) | ” | ” | ” | ” |
| Cla002569 | 3497297..3498866 (-) | pectinesterase/pectinesterase inhibitor | Ripening | Pectinesterase activity | integral to membrane/plasma membrane |
| Cla002572 | 3521685..3524206 (-) | BT1 (BTB AND TAZ DOMAIN PROTEIN 1); protein binding / transcription regulator | Response to carbohydrate stimulus/embryo sac development | transcription regulator activity/protein binding | Nucleus/cytoplasm |
| Cla002573 | 3537784..3539783 (-) | Pectate lyase family protein | cell wall modification during multidimensional cell growth | pectate lyase activity/metal ion binding | Plasma membrane/extra cellular |
| Cla002574 | 3556670..3563276 (+) | Ufm1-specific protease | modification-dependent protein catabolic process | small conjugating protein-specific protease activity | - |
| Cla002575 | 3565013..3568055 (+) | Pentatricopeptide repeat-containing protein | response to oxidative stress | - | Chloroplast |
| Cla002576 | 3574247..3577168 (+) | Programmed cell death protein | cell aging/negative regulation of transcription | protein binding | Nucleus/cytoplasm |
| Cla002578 | 3578921..3586492 (+) | Tetratricopeptide repeat-containing protein | - | Binding | - |
| Cla002579 | 3589309..3595584 (+) | ARP; DNA-(apurinic or apyrimidinic site) lyase | transcription from RNA polymerase II promoter | transcription corepressor activity/DNA binding | Nucleus/cytoplasm/ribosome |
| Cla002580 | 3599536..3603963 (+) | RELATIVE OF EARLY FLOWERING 6 | multicellular organismal development | transcription repressor activity/transcription factor activity | Nucleus/nucleolus/cytoplasm |
| Cla002581 | 3605078..3607207 (+) | Zinc finger protein | vegetative to reproductive phase transition | DNA binding/protein binding | Nucleus/intracellular |
| Cla002582 | 3608887..3613064 (+) | zinc finger (CCCH-type) family protein | carbohydrate transport | RNA/DNA binding | nucleus |
| Cla002583 | 3617281..3618671 (+) | Phosphopentothenoylcysteine decarboxylase | nucleotide biosynthetic process | Protein/FMN binding | Nucleus/cytoplasm |
| Cla002584 | 3620005..3621328 (+) | GDSL-motif lipase/hydrolase family protein | lipid catabolic process | hydrolase activity, acting on glycosyl bonds | Vacuole/cell wall |
| Cla002585 | 3622527..3623899 (-) | zinc finger (C3HC4-type RING finger) family protein | - | zinc ion binding/protein binding | - |
| Cla002586 | 3629716..3633550 (+) | B3 domain-containing protein | regulation of transcription, DNA-dependent | DNA binding | Nucleus |
| Cla002587 | 3634450..3639598 (-) | EMBRYO DEFECTIVE regulator | protein amino acid dephosphorylation | phosphoprotein phosphatase activity | protein serine/threonine phosphatase complex |
| Cla002589 | 3644965..3646162 (+) | GLUCOSE HYPERSENSITIVE 1 | translation | structural constituent of ribosome | Intracellular/ribosome |
| Cla002590 | 3654034..3660212 (+) | 26S proteasome regulatory subunit/ ABC1 family protein | embryonic development ending in seed dormancy | ATP/protein binding | Ubiquitous |
| Cla002591 | 3662188..3663784 (+) | ATP binding / protein kinase | protein amino acid phosphorylation | ATP binding/kinase activity | - |
| Cla002593 | 3668867..3671252 (-) | NUCLEOSIDE DIPHOSPHATE KINASE 2); ATP binding / nucleoside diphosphate kinase/ protein binding | regulation of epidermis development/ GTP biosynthetic process | nucleoside diphosphate kinase activity/transferase activity | Ubiquitous |
| Cla002594 | 3674982..3677063 (+) | Salutaridinol 7-O-acetyltransferase | alkaloid metabolic process | transferase activity, transferring acyl groups other than amino-acyl groups | - |
| Cla002595 | 3678591..3679382 (+) | BAHD acyltransferase | alkaloid metabolic process | acyltransferase activity | - |
| Cla002597 | 3700711..3702880 (+) | Glyceraldehyde-3-phosphate dehydrogenase | fruit development/ glucose metabolic process | catalytic activity | Ubiquitous |
| Cla019343 | 4390396..4396368 (-) | Ankyrin repeat family protein | Response to salt stress | - | Integral to membrane |
| Cla019345 | 4421513..4426340 (-) | Ankyrin repeat family protein | Response to salt stress | - | Integral to membrane |
| Cla019347 | 4445768..4448122 (-) | esterase/lipase/thioesterase | lipid metabolic process | lysophospholipase activity | - |
| Cla019351 | 4502519..4507503 (+) | Ankyrin repeat family protein | - | - | - |
| Cla019354 | 4528705..4532520 (+) | Ankyrin repeat family protein | - | nucleic acid binding | - |
| Cla019356 | 4546903..4547346 (+) | Stigma-specific Stig1 family protein | - | - | - |
| Cla019357 | 4567177..4567740 (+) | Seed protein AmA1 | Pathogenesis | - | Extracellular region |
| Cla019359 | 4576897..4578815 (+) | pectate lyase family protein | cell wall modification during multidimensional cell growth | metal ion binding/lyase activity | plasma membrane |
| Cla019362 | 4598798..4599199 (-) | OTU-like cysteine protease family protein | - | - | - |
| Cla019364 | 4609587..4614243 (+) | ankyrin repeat family protein | - | - | - |
| Cla019365 | 4617007..4617384 (+) | ankyrin repeat family protein | - | - |  |
| Cla019366 | 4630562..4633715 (+) | aspartyl protease family protein | lipid metabolic process | aspartic-type endopeptidase activity | Mitochondria/vacuole |
| Cla019367 | 4634062..4635372 (-) | F-box family protein | - | - | - |
| Cla019368 | 4642202..4643910 (-) | Aminomethyltransferase | response to cadmium ion | aminomethyltransferase activity | mitochondrial inner membrane |
| Cla019371 | 4663056..4666785 (-) | SKS4 (SKU5 Similar 4); copper ion binding / oxidoreductase | lignin catabolic process | L-ascorbate oxidase activity/ copper ion binding | anchored to plasma membrane |
| Cla019372 | 4668165..4670831 (-) | 5'-tyrosyl-DNA phosphodiesterase/hydrolase | double-strand break repair | protein binding/zinc ion binding | PML body/nucleus |
| Cla019373 | 4673347..4676634 (-) | catalytic/ cation binding / hydrolase | carbohydrate metabolic process | hydrolase activity, acting on glycosyl bonds | Plasmodesmata/ anchored to membrane |
| Cla019374 | 4679948..4682049 (-) | APR1 (APS REDUCTASE 1); adenylyl-sulfate reductase | oxidation reduction/ cellular amino acid biosynthetic process | transferase activity/ oxidoreductase activity | Chloroplast stroma/cytoplasm |
